# Rhabdomyolysis after lamotrigine overdose: a case report and review of the literature

**DOI:** 10.1186/s12991-016-0093-3

**Published:** 2016-02-24

**Authors:** Sokratis E. Karaoulanis, Markos Syngelakis, Konstantinos Fokas

**Affiliations:** First Psychiatric Department, Aristotle University of Thessaloniki, Thessaloníki, Greece

**Keywords:** Lamotrigine, Overdose, Rhabdomyolysis

## Abstract

**Background:**

Lamotrigine is an effective anticonvulsant drug that has also been demonstrated to be effective in the treatment of bipolar disorder. We report a case of rhabdomyolysis after intentional overdose in a woman aged 48.

**Case presentation:**

A 48-year-old female presented to the emergency department after an acute ingestion of 6 g of lamotrigine. The patient suffered from bipolar disorder, and she was taking lamotrigine and olanzapine. At that point, she had a major depressive episode, and she wanted to commit suicide. Activated charcoal was administered in the emergency department. Her vital signs were still normal, and she entered the Medical clinic, where she had been there for 2 days in a good condition. The hematological and biochemical results were normal. On the fourth day, the levels of creatine phosphokinase (CPK) showed remarkable increase (2500 IU/ml). Fluid and bicarbonate intravenous administration was performed, and CPK levels returned to normal after 3 days.

**Conclusion:**

The majority of patients exposed to lamotrigine in overdose experienced no toxic clinical effects. The most common clinical effects are drowsiness and lethargy, vomiting, nausea, ataxia, dizziness/vertigo, and tachycardia. In this case report, the patient was alert and did not have any serious complications, except for mild rhabdomyolysis, which was the main consequence of lamotrigine overdose.

## Background

Antiepileptic drugs are being used across a wider number of indications, including bipolar affective disorders and other psychiatric diseases. Carbamazepine, valproate, and lamotrigine have demonstrated efficacy in the treatment of acute mania and are capable of preventing relapse in patients with bipolar disorder. In view of all these factors, there has been more widespread use of antiepileptic drugs in patients with underlying psychiatric disorders, often as an unlicensed indication. This patient group is at substantially increased risk of self poisoning.

We present the case of mild rhabdomyolysis that was caused by lamotrigine overdose in a patient suffering from bipolar disorder.

## Case presentation

A 48-year-old female patient presented to the emergency department after an acute ingestion of 30 tablets of 200 mg lamotrigine. The patient suffered from bipolar disorder, and she was also taking olanzapine and oxcarbazepine. Nevertheless, she admitted that she stopped taking her medication 2 months ago. She said that lamotrigine was the only drug that she had ingested. An empty box containing 30 lamotrigine pills was found next to her bed. The patient was alone when she swallowed the pills. Her daughter told us that her mother had been very sad for a period of 10 days because her son had died because of heroin overdose. Afterward, her mother told her that there was no reason for her to live and to continue taking her medication. Consequently, it seems more possible that the patient did not take any other medication. A toxicologic analysis would have been useful in this case, but unfortunately it was never done.

There was no history of illicit drug use or alcoholism, apart from smoking.

The patient was alert and her blood pressure, pulse rate, and temperature were within normal limits. The neurologic examination disclosed no focal findings.

Gastric lavage with activated charcoal was administered. Hematological and biochemical examinations, including liver function tests, were normal. Her electrocardiogram did not reveal any abnormalities. Then, the patient was admitted to the Medical Clinic, in order to ensure the early detection of any side effects of the high dose of lamotrigine. She remained at the Medical Clinic for 2 days. On the third day, as long as she did not face any problem (the blood and biochemical studies were normal), she was transferred to the Psychiatric Clinic.

The biochemical studies which were carried out in the Psychiatric Clinic showed that the value of creatine phosphokinase (CPK) was 620 IU/ml (normal 24-204 IU/ml). The following day CPK levels reached 2500 IU/ml.

Fluid and bicarbonate intravenous administration was performed, and 3 days later, CPK returned to normal levels.

She was depressed and wanted to commit suicide because of the death of her son. She was suffering from bipolar disorder but did not comply with her pharmacological treatment.

## Review of the literature

The most common clinical effects arising from lamotrigine overdose are drowsiness and lethargy, vomiting, nausea, ataxia, nystagmus, dizziness/vertigo, and tachycardia. Major clinical effects, which are rare, include coma, seizures, respiratory depression, cardiovascular shock, and multi-organ systemic allergic-type reaction [1–11]. In all these case reports, lamotrigine was combined with other drugs. However, Moore et al. reported a case series of patients with lamotrigine only overdose, and they had found that the consequences were seizures, hypertension, tachycardia, tachypnea, altered mental status, myoclonus, and QRS prolongation [10]. In two cases, the patients died, but in one case, there was a multiple drug overdose (lamotrigine, ethanol, thioridazine, carbamazepine, and paroxetine) (Table 1) [11, 12].

**Table 1:** Case reports involving lamotrigine overdose

Case report	Amount of lamotrigine	Disease	Age	Other drugs	Clinical presentation
[16]	4.1 g	Epilepsy	42 year	–	Status epilepticus, ataxia,
[19]	2.7 g	Bipolar disorder	49 year	–	Anticonvulsant hypersensitivity syndrome
[5]	1350 mg	Epilepsy	26 year	–	Ataxia, nystagmus
[15]	800 mg	No disease	2 year	–	Generalized seizures, ataxia
[13]	43 mg/kg		2 year	–	Generalized seizures
[14]	Unknown	No disease	19 month	–	Generalized seizures
[17]	32 g	Epilepsy	29 month	Pregabalin	Reduced levels of consciousness, seizures
[18]	9.2 g	Bipolar disorder	23 year	Citalopram, chlorpheniramine	Reduced levels of consciousness, ECG abnormalities
[8]	4.5 g	Epilepsy	32 year	Ethanol, clonazepam	Ataxia, rotary nystagmus
[22]	2 g	Epilepsy	17 year	Gabapentin	Reduced levels of consciousness, dysarthria, motor incoordination
[9]	600 mg	Epilepsy	31 year	Topiramate, carbamazepine	Nystagmus
[11]	39 mg/L	Epilepsy, depression	21 year	Ethanol, thioridazine, carbamazepine, paroxetine	Death
[20]	Unknown	Bipolar disorder	29 year	Ethanol	Coma, seizures, mild rhabdomyolysis
[21]	Unknown	Epilepsy	55 year	Valproic	Stupor
[6]	7.5 g		48 year	–	Status epilepticus, cardiovascular collapse
[7]					
[12]	4 g	Bipolar disorder	19 year	–	Seizures, respiratory arrest, complete heart block, death
[23]	4 g	Bipolar disorder, ADHD	17 year	Bupropion	Seizures, cardiovascular collapse

The outcome of lamotrigine overdose depends on a variety of factors, like the age, sex, the medical disease, and the simultaneous ingestion of other medications. The age is very important for the clinical outcome of lamotrigine overdose. It seems that in children, the ingestion of high amount of lamotrigine causes generalized seizures [13–15]. In adults, seizures are reported [16], but not so often like in children.

Another important factor for the clinical picture of lamotrigine overdose is the disease of the patient who was taking lamotrigine. The majority of patients suffered from epilepsy. In these cases, lamotrigine, usually, caused seizures [16, 17]. On the contrary, in patients with bipolar disorder, seizures might not be so frequent result [18, 19]. In one case report, a bipolar patient who co-ingested lamotrigine and ethanol developed epileptic seizures [20]. However, it is noteworthy that a bipolar patient presented with anticonvulsant hypersensitivity syndrome (including symptoms such as fever, rash, and hepatitis) after lamotrigine overdose [19].

The combination of lamotrigine with other medications must be taken into account in the cases of lamotrigine overdose. It is combined, usually, with other antiepileptic drugs, with antidepressant drugs and with antipsychotic drugs. In these cases, the level of consciousness is disturbed (reduced level of consciousness, stupor, coma, etc.) [17, 18, 20–22], and cardiovascular collapse may be present [23] Concerning the sex, women are more likely to ingest large amounts of lamotrigine than men [24].

The patient, who was presented above, ingested 6 g of lamotrigine. This is one of the largest amounts of lamotrigine ingested alone, ever reported. This patient developed only mild rhabdomyolysis without any other clinical outcome. Mild rhabdomyolysis is also reported in another case report [20], in which the amount of lamotrigine was unknown and the patient presented with epileptic seizures. Consequently, the authors did not know whether rhabdomyolysis was primary or the result of seizures. In our case, rhabdomyolysis appeared without seizures. Therefore, it seems that mild rhabdomyolysis is the primary outcome of lamotrigine overdose.

In this case, the pathogenic mechanism of rhabdomyolysis is not clear. It could be hypothesized that the overdose of lamotrigine led to rhabdomyolysis due to a genetic predisposition. For example, it was found that patients with virally induced rhabdomyolysis had malignant hyperthermia susceptibility (MHS)-associated.

RYR1 mutations [25, 26]. Therefore, in our case maybe the combination of environmental and genetic factors caused rhabdomyolysis [27].

In general, the ingestion of 6 g of lamotrigine did not cause any serious clinical effect on our patient. This is an important factor for patients with bipolar disorder who are prone to suicidal ideation and commitment of suicide—because these patients might try to commit suicide by taking excessive amounts of medications which are given to them by doctors in order to treat their problem. In addition, lamotrigine protects bipolar patients from the influence of depressive episodes, which are the cause of suicidal ideation. Lithium is another agent used in bipolar disorder, which has proven to be an effective deterrent against the suicidal tendency of these patients. However, lithium has serious adverse effects which limit its use. On the contrary, lamotrigine, when it is titrated slowly to avoid Stevens Johnson syndrome, seems to be a drug without any serious adverse effects. It is noteworthy that lamotrigine has been found to be one of the safest antiepileptic drugs in pregnancy [28].

## Conclusions

In conclusion, mild rhabdomyolysis seems to be a primary effect of lamotrigine overdose, and lamotrigine appears to be quite safe in the case of overdose, especially when it is not combined with other medications.

## Consent

Written informed consent was obtained from the patient for publication of this case report.
